# Upadacitinib for Patients with Rheumatoid Arthritis: A Comprehensive Review

**DOI:** 10.3390/jcm12051734

**Published:** 2023-02-21

**Authors:** Raimon Sanmartí, Hèctor Corominas

**Affiliations:** 1 Arthritis Unit Rheumatology Service, Hospital Clinic of Barcelona and Institut d’Investigacions Biomèdiques August Pi Sunyer (IDIBAPS), 08036 Barcelona, Spain; 2 Servei de Reumatologia, Hospital Universitari de Sant Pau, 08025 Barcelona, Spain; 3 Servei de Reumatologia, Hospital Dos de Maig, 08025 Barcelona, Spain

**Keywords:** Janus kinase inhibitors, upadacitinib, rheumatoid arthritis, efficacy, safety, review

## Abstract

Upadacitinib is a selective and reversible Janus kinase (JAK) inhibitor recently approved by the European Medicine Agency and the Food and Drug Administration for the treatment of rheumatoid arthritis (RA) at a dose of 15 mg/day. We present the chemical structure and mechanism of action of upadacitinib together with a comprehensive review of the efficacy of this drug in RA based on the SELECT clinical trial program and its safety profile. Its role in the management and therapeutic strategy of RA is also discussed. Upadacitinib in the different clinical trials has shown similar rates of clinical response, including the remission rates, regardless of the population analyzed (methotrexate-naïve, methotrexate-failure or biologic failure). In a head-to-head randomized clinical trial, upadacitinib plus methotrexate was superior to adalimumab when given on background methotrexate (MTX) in patients who have experienced an inadequate response to MTX. Upadacitinib also demonstrated superiority over abatacept in patients with RA after failure to previous biologic drugs. The safety profile of upadacitinib is generally consistent with those observed with biological or other JAK inhibitors.

## 1. Introduction

The management of rheumatoid arthritis (RA) has dramatically evolved in the last two decades due to more efficacious therapeutic strategies, the early introduction of antirheumatic drugs and the treat-to-target approach. The development of new drugs, such as biologic (b) disease-modifying antirheumatic drugs (DMARDs), together with a better use of traditional synthetic drugs such as methotrexate, has contributed to a significant improvement in the management of this disease, with better outcomes, including high rates of remission, less radiological damage and a better quality of life [1]. Therefore, RA is currently a treatable disease, although unfortunately there is no definitive cure and not all patients have a satisfactory response to the current therapies. Although several complementary and alternative medicine options have also been explored, currently there is no high-quality evidence to support their use in patients with RA [2].

Janus kinase (JAK) inhibitors have emerged in recent years as a new family of drugs with proven efficacy in several diseases. The inhibition of the JAK/signal transducer and activator of transcription (STAT) pathway by these small molecules, blocking the intracellular signaling mediated by several proinflammatory cytokines, is a new mechanism of action; it differs from the action produced by biological drugs that specifically block a unique cytokine at the extracellular level [3]. This new group of molecules has been investigated in different diseases, including solid malignancies, hematological disorders and different immune-mediated diseases, including RA. At the present time, four JAK inhibitors (JAKi) have been approved by the European Medicine Agency (EMA) for the treatment of RA: tofacitinib, baricitinib and, more recently, upadacitinib and filgotinib; tofacitinib, baricitinib and upadacitinib have been also approved by the US Food and Drug Administration (FDA) for this indication. Tofacitinib inhibits JAK1 and JAK3 and baricitinib inhibits JAK1 and JAK2 [4]. Upadacitinib and filgotinib preferentially inhibit the activity of JAK1 [4,5,6]. These small molecules have demonstrated their efficacy in treating RA in different populations, with a significant improvement not only in the signs and symptoms of RA but also in reducing the radiographic progression of the disease and making a significant impact on patient-reported outcomes (PROs), including pain and the quality of life [7].

JAK inhibitors are recommended for treating RA at the same level as bDMARDs in the therapeutic strategy of RA [8], although in most cases they are still introduced after a failure to use biological drugs. Some advantages have emerged in comparison with biological drugs, such as the oral route and their efficacy as monotherapy (i.e., without concomitant conventional synthetic (cs) DMARDs, notably methotrexate (MTX)) [9]. The safety profile of these drugs has also been carefully evaluated and the rates of various adverse events seem to be similar to those observed with biological drugs, except for a few safety risks reported more frequently with JAKi, such us herpes zoster and blood creatine phosphokinase. Some potential concerns, e.g., cardiovascular events, thrombosis and malignancy, will have to be the subject of long-term pharmacovigilance in observational studies [10].

Upadacitinib preferentially inhibits JAK1. New drug application for the treatment of RA was submitted to the FDA for upadacitinib in December 2018 and currently it is approved by the FDA (August 2019) and the EMA (December 2019) for the treatment of RA at a dose of 15 mg/day. Marketing authorization for treating psoriatic arthritis (FDA and EMA in 2021), ankylosing spondylitis (EMA 2021 and FDA 2022), atopic dermatitis (EMA in 2021 and FDA in 2022), ulcerative colitis (EMA and FDA in 2022) and active non-radiographic axial spondyloarthritis (EMA and FDA 2022) have also been granted. In the present review, we present the chemical structure and mechanism of action of upadacitinib together with a comprehensive review of the efficacy of this drug in RA based on the SELECT clinical trial program and its safety profile, including information from indirect comparisons and real-world studies and contextualized within the regulatory requirements and clinical needs framework. Its role in the management and therapeutic strategy of RA is also discussed.

## 2. Pharmacodynamics

Our better understanding of the pathogenesis of RA through the recognition of key cells and cytokines has led to the development of targeted DMARDs [11]. Many cytokines are involved in the pathogenesis of RA, including tumor necrosis factor (TNF)-α, interferon-γ, interleukin (IL)-1, IL-2, IL-6, IL-8 and IL-17 [11]. Janus kinases (JAKs) are intracellular enzymes that transmit cytokine or growth factor signals involved in a broad range of cellular processes, including inflammatory responses, hematopoiesis and immune surveillance [5]. The JAK family of enzymes contains four members, JAK1, JAK2, JAK3 and TYK2, that work in pairs to phosphorylate and activate signal transducers and activators of transcription (STATs) [5]. This phosphorylation in turn modulates gene expression and cellular function [5]. JAK1 is important in inflammatory cytokine signals, while JAK2 is important for red blood cell maturation and JAK3 signals play a role in immune surveillance and lymphocyte function [5].

JAKi block the enzyme activity of JAK, thereby preventing cytokine signal transduction and cytokine action, and are effective RA therapeutics [11,12]. Upadacitinib was developed to address the hypothesis of whether greater JAK1 selectivity over other JAK family members will translate into a more favorable benefit-risk profile [13]. Upadacitinib selectively targets JAK1-dependent cytokines such as IL-6 and interferon-γ while reducing effects on reticulocytes and natural killer cells [13].

## 3. Chemical Structure and Pharmacokinetics

**Figure 1:**
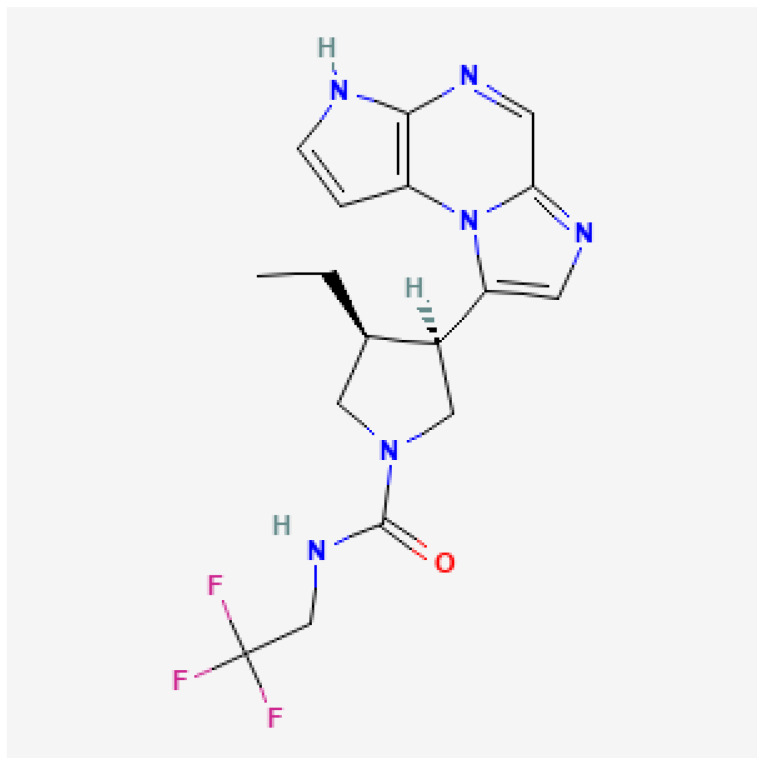
Chemical structure of upadacitinib. Available at [14], Reproduce with permission from the National Library of Medicine.

The International Union of Pure and Applied Chemistry’s (IUPAC) name for upadacitinib is (3*S*,4*R*)-3-ethyl-4-(1,5,7,10-tetrazatricyclo[7.3.0.0^2,6^]dodeca-2(6),3,7,9,11-pentaen-12-yl)-*N*-(2,2,2-trifluoroethyl)pyrrolidine-1-carboxamide (Figure 1) and the molecular formula is C_17_H_19_F_3_N_6_O [14]. The molecular weight is 380.4.

The pharmacokinetic characteristics of upadacitinib have been evaluated in several studies with healthy volunteers and patients with RA using the immediate-release and extended-release formulation. The latter allows a once-daily administration, has been used during the phase III program and was the formulation finally marketed.

The main pharmacokinetic characteristics of the extended-release formulation and the role of intrinsic factors such as ethnicity, age and hepatic and renal impairment in the pharmacokinetics of upadacitinib are summarized in Table 1.

Upadacitinib is a substrate of the isoenzyme CYP3A4 of cytochrome P450, therefore its metabolism could be affected by strong inhibitors or inducers of this isoenzyme. The administration of ketoconazole, a strong inhibitor of CYP3A4, increased the C_max_ and AUC_∞_ by 70% and 75%, respectively [15]. Therefore, upadacitinib 15 mg once daily should be used with caution in patients receiving chronic treatment with strong CYP3A4 inhibitors and alternatives to strong CYP3A4 inhibitor medications should be considered when used in the long term [5]. The administration of rifampicin, a strong inducer of CYP3A4, decreased the C_max_ by 50% and the AUC_∞_ by 60%. Therefore, patients should be monitored for changes in disease activity if upadacitinib is coadministered with strong CYP3A4 inducers.

The CYP2D6 phenotype (i.e., poor or extensive metabolizer) did not affect the apparent oral clearance of upadacitinib; thus, medications that are strong inhibitors of CYP2D6 are not expected to interact to a clinically relevant extent with upadacitinib [15].

Methotrexate and pH-modifying medicinal products (e.g., antacids or proton pump inhibitors) have no effect on upadacitinib plasma exposure [5,15]. Coadministration of upadacitinib with rosuvastatin or atorvastatin has no relevant effects on the exposure to these drugs, therefore no dose adjustments are required [15]. Upadacitinib has no relevant effects on plasma exposure to ethinylestradiol, levonorgestrel, methotrexate or medicinal products that are substrates for metabolism by CYP1A2, CYP2B6, CYP2C9, CYP2C19 or CYP2D6 [5,15].

## 4. Efficacy

### 4.1. Phase 2 Studies with Upadacitinib and Dose Selection for the Phase 3 Program

Two phase 2b dose-ranging studies were conducted with the twice-daily dosing of an immediate-release formulation of upadacitinib: the BALANCE I and BALANCE II trials [16,17]. The BALANCE I was undertaken in patients with RA and an inadequate response to at least one anti-TNF. It tested doses of upadacitinib of 3, 6, 12 and 18 mg twice daily in a randomized double-blind placebo-controlled design [16]. The proportion of American College of Rheumatology 20% improvement criteria (ACR20 responders), the primary endpoint, showed a dose–response relationship, with all doses being superior to placebo at week 12. Similarly designed, the BALANCE II evaluated the efficacy and safety of upadacitinib at doses of 3, 6, 12 and 18 mg twice daily and 24 mg once daily in patients with RA and an inadequate response to methotrexate [17]. This study also showed a significant dose–response relationship, but only doses of 6 and 12 mg twice daily and 24 mg once daily were significantly superior to placebo regarding the proportion of patients achieving an ACR20 response at week 12.

An exposure–response modeling analysis of the results of these phase 2 studies using plasma exposure to upadacitinib and efficacy results as evaluated with ACR response criteria suggested that plasma concentrations associated with 15 and 30 mg once daily doses were predicted to achieve a plateau of response across RA subpopulations [18]. This was the basis for selection of the 15 and 30 mg doses of upadacitinib extended release for evaluation in the phase 3 trial program.

### 4.2. The SELECT Phase 3 Trial Program with Upadacitinib in Patients with RA

The phase 3 clinical trial program for the evaluation of upadacitinib in patients with RA was designed to meet the regulatory requirements of the US FDA [19] and those of the EMA [20]. The EMA guideline for evaluating medicinal products for the treatment of rheumatoid arthritis (RA) recommends performing trials in two distinct clinical settings: patients with an inadequate response to one or more DMARDs and DMARD-naïve patients. The new drug could receive the indication either as monotherapy or in combination with methotrexate (MTX) or another csDMARD [20].

The SELECT trial program for evaluating upadacitinib in patients with RA included six global clinical trials (Table 2) [21,22,23,24,25,26]. Five trials were conducted in patients with an inadequate response (IR) to MTX or other DMARDs. Of them, in four trials, upadacitinib was tested in combination with MTX/csDMARD background therapy: two trials were placebo-controlled trials without active comparators (SELECT-NEXT in a csDMARD-inadequate response (IR) population [21] and SELECT-BEYOND in a bDMARD-IR population [22]) and two trials included an active comparator (SELECT-COMPARE in a MTX-IR population [23] and SELECT-CHOICE in a bDMARD-IR population [24]). Another trial in patients with an inadequate response to MTX was conducted with upadacitinib monotherapy: the SELECT-MONOTHERAPY [25]. Finally, a single trial, the SELECT-EARLY trial [26], was conducted in MTX-naïve patients where upadacitinib was evaluated as monotherapy.

All SELECT programs included patients with adult-onset RA and were multicenter, randomized and double-blind and, with the exception of active comparator trials in patients with an inadequate response to DMARDs, all trials evaluated doses of 15 and 30 mg once-daily (QD) of upadacitinib (Table 2). The study duration varied across studies, but the primary endpoint was generally established at week 12. All but one tested a superiority hypothesis; the SELECT-CHOICE tested the noninferiority of upadacitinib 15 mg QD vs. abatacept and, after its confirmation, the superiority of upadacitinib over abatacept was also tested as a confirmatory endpoint.

FDA and EMA differ in the recommended primary endpoint for drugs intended for the treatment of RA. While the ACR20 response criteria are an accepted primary efficacy measure to demonstrate a reduction in RA disease activity [19], for the EMA the primary endpoint should reflect a target disease state, that is, remission or low disease activity (LDA) [20]. Consistent with these regulatory requirements, primary efficacy outcomes in the SELECT trial program included two separate primary endpoints, the ACR20 and a disease activity score in 28 joints using C-reactive protein level (DAS28-CRP) measure of remission or LDA; the exception was the noninferiority SELECT-CHOICE trial, which used the mean change from baseline in the DAS28-CRP score as the primary outcome. Secondary confirmatory endpoints consisted of a variety of measures evaluating response, remission, LDA and patient-reported outcomes that provide a multidimensional picture of the impact of upadacitinib in patients with RA. The impact of upadacitinib on structural damage as evaluated with radiographic outcome measures was evaluated in the SELECT-COMPARE and SELECT-EARLY. Table 2 summarizes the key characteristics of the trials included in the SELECT program for RA.

#### 4.2.1. Primary Efficacy Results

##### Upadacitinib in Patients with an Inadequate Response to Methotrexate or Other DMARDs

- Placebo-Controlled Trials

Two placebo-controlled trials with no active comparators confirmed the efficacy of upadacitinib in combination with csDMARDs in patients with an inadequate response to MTX or other DMARDs: SELECT-NEXT [21] and SELECT-BEYOND [22].

The SELECT-NEXT trial was a randomized double-blind placebo-controlled trial that included adult patients with active RA for 3 months or longer who had received csDMARDs for at least 3 months and had an inadequate response to at least one csDMARD; patients with an inadequate response to bDMARD were excluded [21]. It was allowed that up to 20% of the patients had been exposed to one bDMARD, provided that exposure was short or that the patient had discontinued the bDMARD because of intolerance. The study comprised a 12-week double-blind period followed by a double-blind extension of up to 5 years. The study included 661 patients who were randomized to placebo (*n* = 221), upadacitinib 15 mg QD (*n* = 221) or upadacitinib 30 mg QD (*n* = 219). Most patients were female, the mean time since diagnosis was over 7 years and the mean DAS28-CRP at baseline was 5.6. Sixty percent of the patients were receiving MTX and 20% MTX plus another csDMARD. At week 12, both doses of upadacitinib met the primary endpoint of ACR20 with statistically significant differences relative to placebo, which we consider clinically relevant (consistently with other authors [27], we think that a difference of 10% in a binary outcome is the lower limit to consider that a difference is clinically relevant) (Table 3). The results regarding the proportion of patients showing a DAS28-CRP ≤ 3.2, the other primary endpoint, were also statistically significantly different from placebo and, in our view, clinically relevant. There was a rapid onset of action; thus, at week 1, the proportion of patients achieving an ACR20 response was 22% and 28% with upadacitinib 15 mg and 30 mg QD, respectively, which was statistically significantly superior to placebo (9% of ACR20 responses).

SELECT-BEYOND had a similar design to SELECT-NEXT, except that patients in the SELECT-BEYOND had to have a previous inadequate response or intolerance to bDMARDs and in the SELECT-NEXT to csDMARDs [22]. The study has a double-blind extension of up to 5 years. Enrolled patients had a longer disease duration (a mean of 13 years) than in SELECT-NEXT and, importantly, 47% had received one previous bDMARD, 28% had received two and 25% had received three. The results of primary endpoints (i.e., ACR20 response and DAS28-CRP ≤ 3.2 at week 12), shown in Table 3, were similar to those observed in SELECT-NEXT. The similarity of the results between SELECT-BEYOND and SELECT-NEXT is important, since patients in SELECT-BEYOND were more refractory to treatment. It should be noted that patients with an inadequate response to bDMARDs usually show lower response rates than patients with an inadequate response to csDMARDs [28,29]. Moreover, in a subgroup analysis of SELECT-BEYOND, the number and mechanism of action of previous bDMARDs did not modify the ACR20 response at week 12 [22]. Similar to SELECT-NEXT, upadacitinib at either dose was superior to placebo as early as week 1.

- Active-Controlled Trials

To contextualize the efficacy and safety of a new product in the setting of an inadequate response to DMARDs, the EMA requires that at least one of the confirmatory trials include an active comparator [20]. The SELECT program included two active comparator trials in this context: the placebo-controlled SELECT-COMPARE vs. adalimumab [23] and the SELECT-CHOICE vs. abatacept [24].

SELECT-COMPARE was a randomized double-blind placebo-controlled trial conducted in patients with active RA who had an inadequate response to MTX and that is foreseen to have a 10-year follow-up. It was allowed that up to 20% of the patients had been exposed to one bDMARD, provided that exposure was short or that the patient had discontinued the bDMARD because of intolerance. Importantly, the sample was enriched to recruit patients who had 1–3 erosions at baseline, thus allowing for radiographic progression within the timeframe of the study (see below). Patients were randomized to receive upadacitinib 15 mg QD, adalimumab 40 mg every other week or placebo. The primary efficacy endpoints were the achievements of the ACR20 response and DAS28-CRP < 2.6 compared to placebo at week 12. The comparison of upadacitinib with adalimumab was part of the key secondary confirmatory endpoints and included the noninferiority of upadacitinib in terms of ACR50 response rate and DAS28-CRP ≤ 3.2 at week 12 and the superiority according to the ACR50 response rate and the mean change from baseline in pain severity score and health assessment questionnaire disability index (HAQ-DI) score.

The results regarding the ACR20 response in the SELECT-COMPARE were consistent with those in SELECT-NEXT and SELECT-BEYOND, with over two-thirds of the upadacitinib-treated patients achieving an ACR20 response at week 12 (Table 3). At week 12, almost one-third of the patients achieved a DAS28-CRP < 2.6 (remission), with a difference compared with placebo that was statistically significant and, in our view, clinically relevant. Upadacitinib also met the noninferiority comparison to adalimumab in the abovementioned endpoint and subsequently demonstrated its superiority over adalimumab in the achievement of the ACR50 response at week 12 (45% vs. 29%, *p* < 0.001); it also met the multiplicity-adjusted superiority in the mean change from baseline in pain severity score and HAQ-DI score (see below). It is important to note that the responses in adalimumab-treated patients were consistent with those reported in previous trials in patients with an inadequate response to MTX [30,31].

The SELECT-CHOICE trial was a double-blind trial that included adult patients with moderate-to-severe active RA who had an inadequate response to at least one bDMARD [24]. Patients were randomized to receive upadacitinib 15 mg QD or intravenous abatacept at a weight-adjusted dose on day 1, week 2 and every 4 weeks thereafter until week 20. After completing the 24-week double-blind phase, patients could be treated with upadacitinib in an open-label fashion for up to 5 years. The primary endpoint was the mean change from baseline to week 12 in the DAS28-CRP score, which was tested for noninferiority, with a noninferiority margin of 0.6. If noninferiority was demonstrated, the superiority hypothesis was tested. After 12 weeks of treatment, the mean change from baseline in the DAS28-CRP score was −2.52 points among upadacitinib-treated patients and −2.00 points among abatacept-treated patients, for an estimated treatment difference of −0.52 (95% CI, −0.69 to −0.35), meeting the noninferiority hypothesis and subsequently meeting the superiority hypothesis (Table 3).

- Upadacitinib as Monotherapy in Patients with Inadequate Response to csDMARDs

Despite guidelines on the management of RA recommending that, when indicated, bDMARDs should be used in combination with at least a csDMARD [8], approximately 25–30% of the patients who are under treatment with a bDMARD were receiving it as monotherapy [32,33]. The reasons for this practice include tolerability or contraindications to csDMARDs or the presence of comorbidities [33,34]. The aim of SELECT-MONOTHERAPY was to evaluate the efficacy and safety of upadacitinib monotherapy in patients with an inadequate response to MTX [25]. The trial included patients with active RA who were receiving MTX for at least 3 months and were on a stable dose for at least 4 weeks before study entry. Patients were randomized to receive upadacitinib 15 mg or 30 mg QD or to continue with their prior dose of MTX; subjects in the latter group were re-randomized to upadacitinib 15 mg and 30 mg at week 14. The study has a blinded extension of up to 5 years. The primary endpoints were the ACR20 response rate and the proportion of patients achieving a DAS28-CRP ≤ 3.2 at week 14. The primary efficacy results were similar to those reported for the combination therapy described above, with over two-thirds of the patients achieving an ACR20 response with both doses of upadacitinib and differences with continued-MTX that were statistically significant and, in our view, clinically relevant (Table 3). The results regarding LDA as evaluated with the DAS28-CRP were also similar to those reported with the combination therapy, although numerically the proportion of patients meeting this endpoint was slightly higher with upadacitinib 30 mg QD than with a dose of 15 mg QD (Table 3).

##### Upadacitinib in Patients Who Are Naïve or Have Limited Exposure to MTX

Upadacitinib was evaluated in this population in the SELECT-EARLY trial [26]. In this trial, patients with active RA and symptoms of RA for ≥6 weeks and who were MTX naïve or who had received MTX for ≤3 weeks were randomly assigned to receive upadacitinib 15 mg or 30 mg QD or weekly MTX (10–20 mg/week) for 48 weeks, followed by an open-label extension of up to 5 years. According to the clinical practice guidelines for the management of RA, in this population, the treatment goal should be remission or at least an LDA state [8,35]. Consistent with these goals, the primary efficacy endpoints in the SELECT-EARLY trial were the ACR50 response rate at week 12 and DAS28-CRP < 2.6 at week 24. Both doses of upadacitinib met the ACR50 response criteria, with a response rate over 50%, rates that were significantly superior to those of placebo (Table 3). Remission, as evaluated with DAS28-CRP at week 24, was also achieved by approximately 50% of the patients with both doses of upadacitinib. We consider that the differences compared with placebo and MTX for both doses of upadacitinib were clinically relevant.

#### 4.2.2. Clinical Remission with Upadacitinib

Treat-to-target should be the strategy when treating patients with RA and, according to clinical practice guidelines, although LDA is a reasonable initial target, clinical remission is the main therapeutic target for patients with RA [8,35].

**Figure 2:**
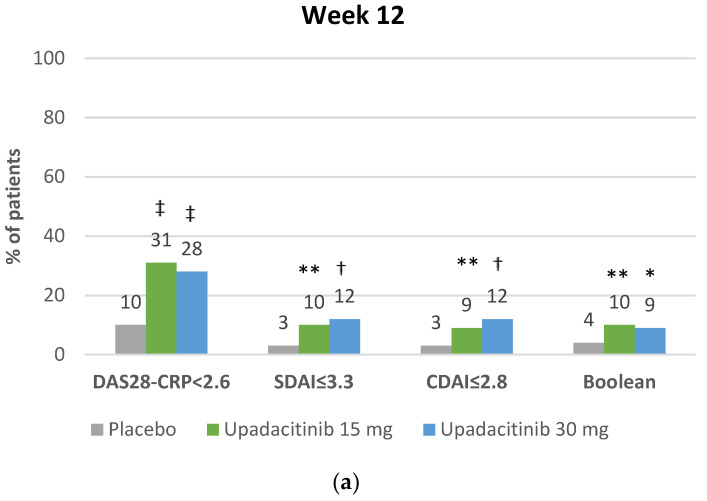

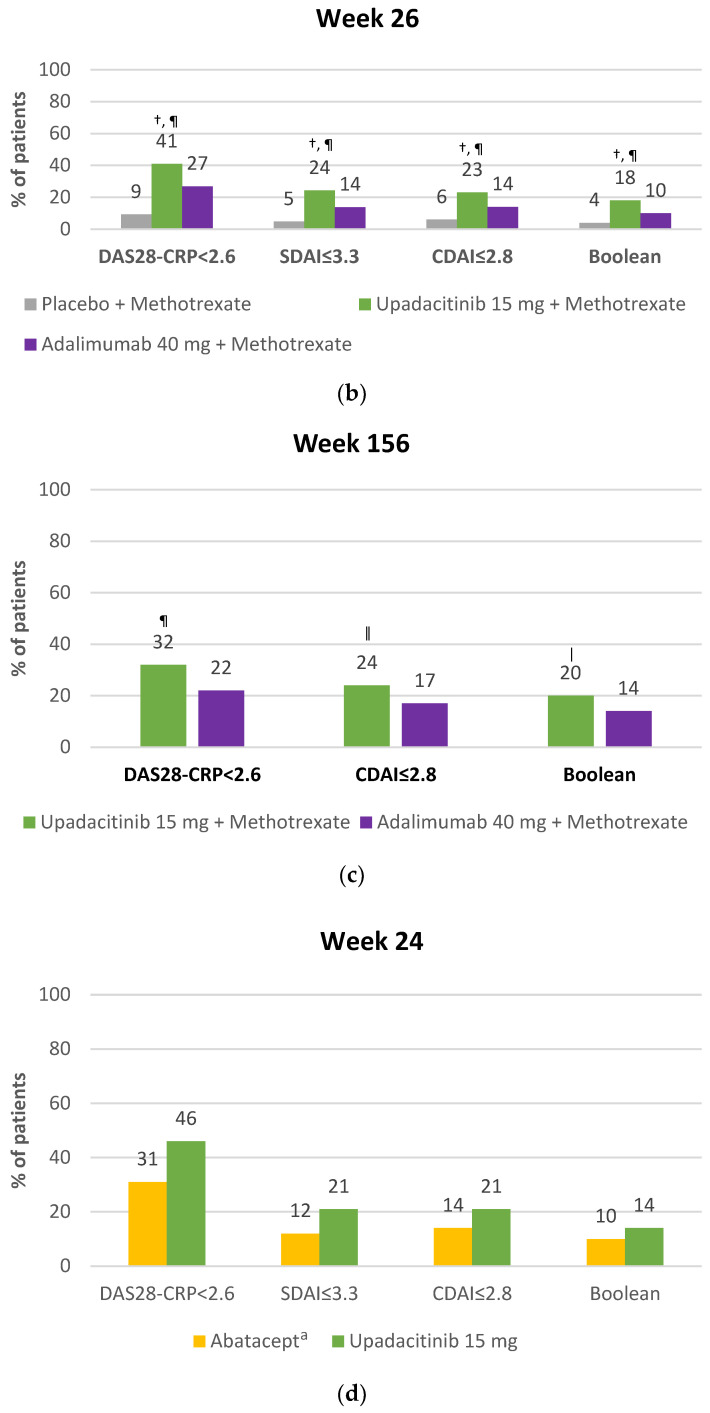

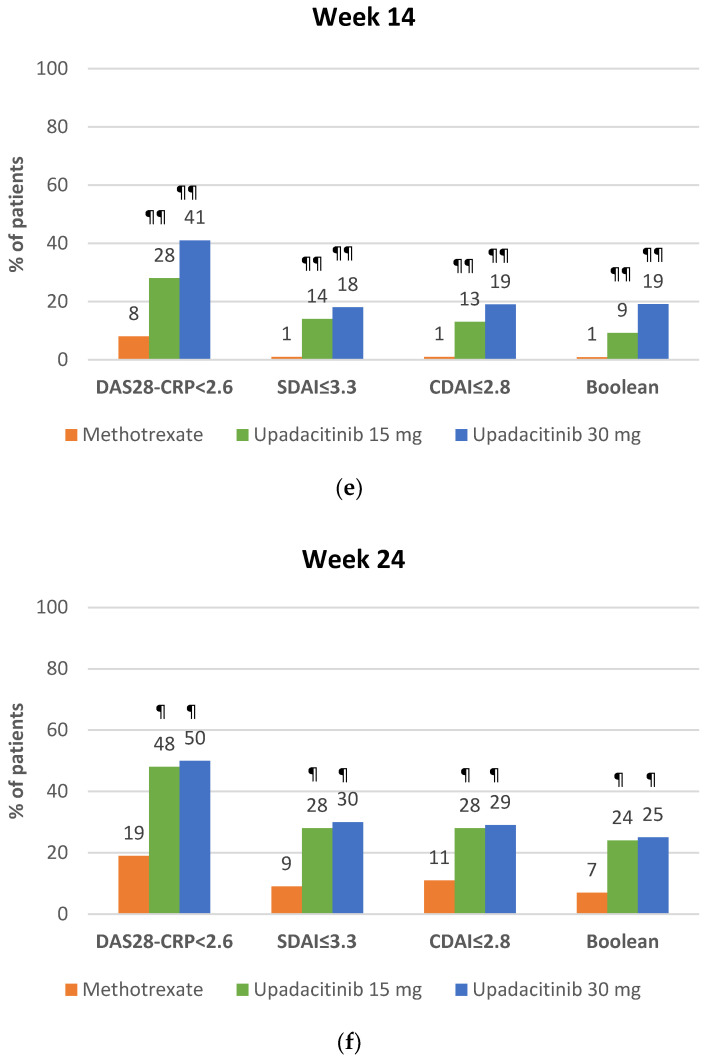
(**a**) Clinical remission with upadacitinib in the SELECT-NEXT trial [21]. * *p* < 0.05 for upadacitinib vs. placebo. ** *p* < 0.01 for upadacitinib vs. placebo. ^†^ *p* < 0.001 for upadacitinib vs. placebo. ^‡^ *p* < 0.0001 for upadacitinib vs. placebo. (**b**) Clinical remission with upadacitinib in the SELECT-COMPARE trial at week 26 [23]. ^†^ *p* < 0.001 upadacitinib vs. placebo. ^¶^ *p* < 0.001 upadacitinib vs. adalimumab. (**c**) Clinical remission with upadacitinib in the SELECT-COMPARE trial at week 156 [36]. ^ǀ^ *p* < 0.05 upadacitinib versus adalimumab. ^ǁ^ *p* < 0.01 upadacitinib versus adalimumab. ^¶^ *p* < 0.001 upadacitinib versus adalimumab. (**d**) Clinical remission with upadacitinib in the SELECT-CHOICE trial [24]. ^a^ No statistical testing was performed. (**e**) Clinical remission with upadacitinib in the SELECT-MONOTHERAPY trial [25]. ^¶¶^ *p* < 0.0001 for upadacitinib versus methotrexate. (**f**) Clinical remission with upadacitinib in the SELECT-EARLY trial [26]. ^¶^ *p* < 0.001 for upadacitinib versus methotrexate. CDAI—clinical disease activity index; DAS28-CRP—disease activity score in 28 joints using C-reactive protein level; SDAI—simplified disease activity index.

The remission rates in the SELECT program according to several definitions are presented in Figure 2a–f. In patients with an inadequate response to other DMARDs, when evaluated with the widely used definition of achieving a DAS28-CRP score of less than 2.6, upadacitinib 15 mg QD in combination with a DMARD background therapy consistently showed a remission rate of over 30% at week 12 [21] and over 40% at week 24–26 [23,24], with differences with placebo that were statistically significant and, in our view, clinically relevant. Importantly, active-controlled trials showed that the remission rate with upadacitinib 15 mg QD was significantly greater compared with adalimumab (nominal *p*-value) and upadacitinib 15 mg QD was superior to abatacept (adjusted for multiplicity *p*-value) in terms of remission. In the SELECT-COMPARE trial, at week 26, 41% of the patients treated with 15 mg QD achieved a DAS28-CRP < 2.6 compared with 27% among adalimumab-treated patients, differences that were also statistically significant and, in our view, clinically relevant (Figure 2b); almost identical results were reported in the SELECT-CHOICE trial, where the proportion of patients who achieved remission at week 24 was 46% with 15 mg QD upadacitinib and 31% with abatacept (Figure 2d).

JAK inhibitors are involved in the interleukin-6 signaling pathway, which is a driver of acute phase inflammatory responses, including the induction of CRP [37]; therefore, it is also important to know the remission rates according to an outcome measure that does not include CRP, such as the clinical disease activity index (CDAI). Using the more stringent criteria of achieving a CDAI ≤ 2.8, remission rates with upadacitinib 15 mg were approximately 10% at week 12 [21] and approximately 20% at week 24–26 [23,24]; differences with placebo were statistically significant and, at week 26, we also consider as clinically relevant [23]. Compared with adalimumab, the proportion of patients achieving a CDAI ≤ 2.8 at week 26 with upadacitinib 15 mg QD was also higher (23% vs. 14%, *p* < 0.001) (Figure 2b) [23]. These rates at week 24 for the comparison of upadacitinib 15 mg QD and abatacept were 21% and 14%, respectively (hypothesis testing not available) (Figure 2d) [24].

ACR/EULAR Boolean remission is achieved when meeting the following four criteria: tender joint count ≤ 1 (based on 28 joints); swollen joint count ≤ 1 (based on 28 joints); CRP ≤ 1 mg/dL and physician global assessment ≤ 1 on a 0–10 scale [38]. This definition of remission is the most stringent criteria used in clinical trials and it is supported by a stronger correlation with lower rates of radiographic progression than other definitions, such as that based on the DAS28-CRP [38]; it is also associated with lower long-term disability [39]. However, when applied to clinical practice, very few patients meet these Boolean-based criteria [40,41]. In two US samples of patients with RA, only 5–6% met the Boolean remission criteria [40]. Similar results were reported in a German sample of ‘real world’ practice patients (7% were remitted with the Boolean criteria, compared with 28% with the DAS28-CRP criteria) [41]. In this context, the Boolean remission rates with upadacitinib 15 mg QD in combination with other DMARDs were relatively high (10% at week 12 [21] and 14–18% at week 24–26 [23,24]) and the difference with placebo at week 26 was not only statistically significant but, in our view, also clinically relevant [23]. Noticeably, the difference with adalimumab was also statistically significant and, for this stringent outcome, we think it could be considered clinically relevant (18% vs. 10%, *p* < 0.001) (Figure 2b) [23]. Upadacitinib showed also significantly greater (nominal *p*-value) remission rates compared with abatacept using the Boolean remission criteria; however, we think that the difference is of doubtful clinical relevance (Figure 2d) [24].

Results for a longer term are available for only the SELECT-COMPARE trial [42]. At week 48, remission rates, regardless of the criteria, were at least maintained compared with those at week 26 [42]. The proportions of patients achieving remission with 15 mg QD upadacitinib and adalimumab were as follows: DAS28-CRP: 38% vs. 28% (*p* < 0.01); SDAI ≤ 3: 25% vs. 17% (*p* < 0.01); CDAI ≤ 2.8: 25% vs. 17% (*p* < 0.01); Boolean remission: 21% vs. 15% (*p* < 0.05) [42]. A recent analysis over 3 years of this long-term extension continues to show higher remission rates with upadacitinib in comparison with adalimumab (Figure 2c) [36].

In patients with inadequate responses to other DMARDs, the results of upadacitinib 15 mg QD monotherapy compared with continuing with MTX (SELECT-MONOTHERAPY) were also consistent with those of the trials evaluating combination therapy, with remission rates at week 14 using the DAS28-CRP criteria of 28% with upadacitinib 15 mg QD and 8% with continued-MTX (*p* < 0.0001) and using the CDAI criteria of 13% vs. 1% (*p* < 0.0001) (Figure 2e) [25]. Similarly, using the Boolean-based remission criteria, the rates were also similar to those reported for combination therapy (at 14 weeks, 9% with upadacitinib 15 mg QD vs. 1% with continued-MTX, *p* < 0.0001) [25].

In patients with RA who were MTX-naïve or had very limited exposure to MTX, the SELECT-EARLY trial showed that the differences in upadacitinib 15 mg QD over MTX were larger than those in more experienced patients (Figure 2f) [26]. Thus, using the most stringent criteria of the Boolean remission, 24% of the upadacitinib-treated patients met this endpoint at week 24 compared with 7% of the MTX-treated patients (*p* < 0.001) [26].

In the SELECT program, upadacitinib 30 mg QD did not seem to provide any relevant additional benefit in terms of remission over the dose of 15 mg QD, with the exception of the SELECT-MONOTHERAPY trial, where the remission rates were numerically higher with the highest dose [25]. However, this benefit was not seen in other SELECT trials, including SELECT-EARLY [26].

#### 4.2.3. Impact of Upadacitinib on Patient-Reported Outcomes

The FDA guidelines for the development of drugs for the treatment of RA include physical function as a key domain for establishing the efficacy of the drug and recommend the HAQ-DI for demonstrating improvement in this domain [19]. Outcomes such as symptoms, especially pain and physical function, are important for patients because they have an impact on their quality of life and daily activities [43,44,45]. In a survey completed by 274 patients from several European countries and the US evaluating patients’ perspectives on remission, the domains most frequently rated as ‘essential’ to characterize a period of remission were as follows: pain (60%), being mobile (52%), physical function (51%), being independent (47%) and fatigue (41%) [46].

In Table 4, we summarize the results of all SELECT trials for those key domains; more specifically, we have included mean changes from baseline in the pain severity, functional assessment of chronic illness therapy-fatigue (FACIT-F) score, the physical component score of the short form-36 health survey (SF-36) and the score of the HAD-DI. Across these four outcome measures, in patients with RA who had an inadequate response to DMARDs, upadacitinib 15 mg in combination with background DMARDs was statistically significantly superior to placebo and as monotherapy was superior to continued-MTX, with differences that were also clinically relevant as judged by comparing the estimated treatment differences with the minimum clinically important difference (i.e., the smallest benefit of value to patients [47]). This was also true for the differences in upadacitinib 15 mg over MTX in patients who were MTX-naïve or had limited exposure to MTX.

In the SELECT-COMPARE, upadacitinib 15 mg achieved significantly greater responses compared with adalimumab at weeks 12 and 26, demonstrating its superiority over adalimumab in HAQ-DI and pain improvements at week 12 (Table 4) [23]. Based on a responder analysis of PROs of this trial (i.e., categorizing patients as responders based on a defined threshold for the specific outcome measure (e.g., the minimum clinically important difference, normative values)), the proportion of patients with clinically relevant improvements at week 12 did not differ between upadacitinib 15 mg QD and adalimumab, but, at weeks 26 and 48, a greater proportion of patients maintained clinically relevant improvements in pain, fatigue and the physical component of the quality of life and physical functioning scale [48]. At week 48, the proportion of patients who maintained clinically relevant changes for upadacitinib 15 mg QD vs. adalimumab were as follows: pain (57% vs. 45%), fatigue (56% vs. 43%), SF36-PCS (59% vs. 42%) and HAQ-DI (60% vs. 42%); all of these differences were statistically significant and, in our view, clinically relevant.

In the SELECT-CHOICE [24], mean changes in fatigue and the physical component of the SF36 were numerically greater with upadacitinib 15 mg QD than with abatacept, but the differences were not large (Table 4).

As was the case for the other outcomes mentioned above, with the exception of SELECT-MONOTHERAPY, the results with upadacitinib 15 mg and upadacitinib 30 mg were also similar regarding PRO (Table 4).

#### 4.2.4. Radiographic Outcomes with Upadacitinib

Structural damage leads to functional disability, which, in contrast to the disability derived from disease activity, is largely irreversible [49]. Thus, the prevention of structural damage evaluated as radiographic progression is the primary goal of therapy [8,35] and a key outcome during the development of drugs for the treatment of RA [19,20].

**Figure 3:**
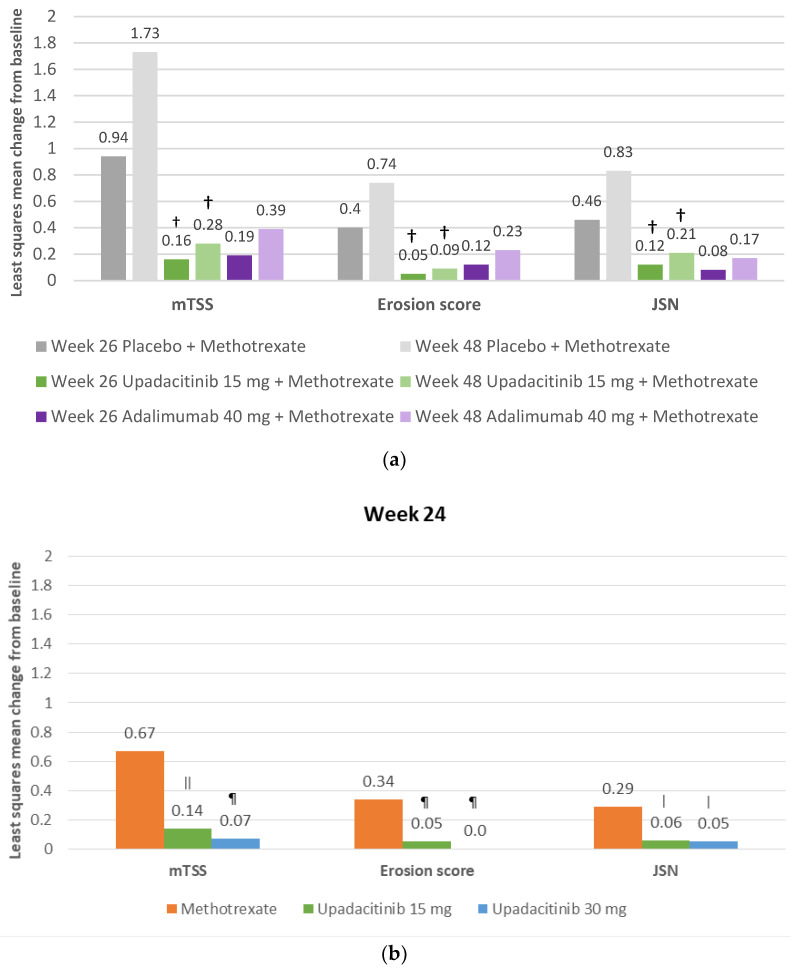
(**a**). Radiographic progression in the SELECT-COMPARE trial at week 26 and 48. ^†^ 0.001 for upadacitinib versus placebo + methotrexate for the comparisons at each timepoint. Adapted and reproduced from Fleischmann RM, Genovese MC, Enejosa JV, Mysler E, Bes-sette L, Peterfy C, Durez P, Ostor A, Li Y, Song IH. Safety and effectiveness of upadacitinib or adalimumab plus methotrex-ate in patients with rheumatoid arthritis over 48 weeks with switch to alternate therapy in patients with insuffi-cient response. Ann Rheum Dis. 2019 Nov;78(11):1454-1462, © 2019, with permission from BMJ Publishing Group Ltd. (**b**). Radiographic progression in the SELECT-EARLY trial. ǀ 0.05 upadacitinib versus methotrexate. ǁ 0.01 upadacitinib versus methotrexate. ¶ 0.001 upadacitinib versus methotrexate. Reproduced with permission from van Vollenhoven R, Takeuchi T, Pangan AL, Friedman A, Mohamed MF, Chen S, Rischmueller M, Blanco R, Xavier RM, Strand V. Efficacy and Safety of Upadacitinib Monotherapy in Methotrex-ate-Naive Patients With Moderately-to-Severely Active Rheumatoid Arthritis (SELECT-EARLY): A Multicenter, Multi-Country, Randomized, Double-Blind, Active Comparator-Controlled Trial. Arthritis Rheumatol. 2020 Oct;72(10):1607-1620, © 2020 The Authors. Arthritis & Rheumatology published by Wiley Periodicals LLC on behalf of American College of Rheumatology. JSN—joint space narrowing; mTTS—modified total Sharp/van der Heijde score.

The effects of upadacitinib on radiographic progression were evaluated in patients with an inadequate response to MTX in the SELECT-COMPARE using an enriched sample and in MTX-naïve patients in the SELECT-EARLY. In the SELECT-COMPARE study, the mean changes from baseline in the van der Heijde modified total Sharp score (mTSS) were statistically significantly lower with 15 mg QD of upadacitinib than with placebo, both at week 26 (0.16 vs. 0.94) and week 48 (0.28 vs. 1.73) (Figure 3a) [42]. Changes with adalimumab were somewhat similar to those with upadacitinib 15 mg QD [42]; at week 48, the proportion of patients with no progression was 86% and 88% for upadacitinib 15 mg and adalimumab, respectively [42]. The erosion score and the joint space narrowing score showed trends similar to those of the mTSS (Figure 3a).

Similarly, in patients with RA who were MTX-naïve, upadacitinib 15 mg QD also significantly reduced radiographic progression compared with MTX at week 24 (Figure 3b).

### 4.3. Comparative Efficacy of Janus Kinase Inhibitors

There are no head-to-head comparisons of upadacitinib with other JAKi. However, several network meta-analyses that provide an indirect comparison of upadacitinib with at least one other JAKi have been published [50,51,52,53,54,55,56]. Most of them evaluated the efficacy of these drugs in patients with an inadequate response to DMARDs, specifically csDMARDs [52,56], bDMARDs [53] or both [50,55]. One of them evaluated monotherapy with JAKi and included studies conducted in patients with an inadequate response to csDMARD and in DMARD-naïve patients [51]. Finally, one network meta-analysis was focused on naïve patients [54]. In several of these network meta-analyses, the number of included studies was very small (i.e., ≤5 studies) [51,53,54] and, as recognized by the authors, heterogeneity—a major threat to meta-analysis—was an issue in several of the meta-analyses [50,51,52,53,54]. In all but one [55] of these meta-analyses, the connectivity of the networks was fairly poor (i.e., a star network) and, in one of the meta-analyses, the selection of the studies was based on two previous reviews [56]; therefore, the results of all of these meta-analyses should be interpreted with great caution, especially with regard to comparative efficacy.

Although rankings based on the surface under the cumulative ranking curve (SUCRA) varied depending on the outcome and the study, based on the 95% credibility intervals provided in the league tables, most of the pairwise comparisons between JAKi were not statistically significant.

### 4.4. Real-World Data with Upadacitinib

With the limitations inherent to observational studies, data from registries and other sources of real-world information may complement the information from randomized clinical trials, providing results on how different strategies perform in clinical practice [57,58]. Although upadacitinib has only recently been marketed, some real-world studies have been reported as congress communications [59,60,61,62].

Using data from a US disease-based registry, Kremer et al. [59] analyzed 300 patients who initiated upadacitinib treatment, 67% of them as a third or higher line of therapy; prior use of a TNFi and JAKi was reported in 79% and 49% of the patients, respectively, and 37% of patients showed high disease activity. Among the initiators who had information on CDAI at 6 months, 35% moved from high to moderate disease activity, 26% moved from high or moderate to LDA, 11% moved from high/moderate/low disease activity to remission and 41% maintained their disease status [59]. Clinically relevant improvements at 6 months as assessed by a responder analysis using the minimal clinically important difference (MCID) were reported in 37% of the patients for the HAQ-DI, 47% for pain and 41% for fatigue [59].

An analysis of another real-world US registry included 1892 patients treated with upadacitinib, 53% as monotherapy and a large proportion who had received prior treatment with bDMARDs (78% and 68% among those treated with monotherapy and combination therapy, respectively) or treatment with any JAKi (50% and 39% among those treated in monotherapy and combination therapy, respectively) [60]. Among the 226 patients with CDAI information at 6 months, 35% were in LDA/remission and 36% showed improvement in disease activity [60].

Two hundred and fifty-two patients treated with upadacitinib 15 mg QD were included in an analysis of a US database, 39% as upadacitinib monotherapy and most of them previously treated with a csDMARD (86%), bDMARD (72%) or a JAKi (48%) [61]. At 3 months, evaluated with the CDAI, 12% were in remission and 43% showed LDA [61]. When analyzed by type of therapy, the proportion of patients who achieved CDAI remission was 16% with monotherapy and 9% with combination therapy and, among those with LDA, it was 50% with monotherapy and 38% with combination therapy [61]. Among the 113 patients who had previously received tofacitinib, 14% achieved CDAI remission and 46% achieved LDA [61].

Finally, the interim results of a German postmarketing authorization noninterventional study have recently been reported [62]. Of the 483 patients included, 61% had previously been treated with b- or targeted-synthetic (ts)DMARDs. Among the 276 patients who completed 6 months of treatment with upadacitinib 15 mg (i.e., an observed case analysis), 25% achieved CDAI remission and 75% LDA. Although no specific data are provided, the authors mentioned that the safety profile of upadacitinib 15 mg was consistent with that observed in the phase 3 studies.

## 5. Safety

An integrated safety analysis was performed using data from five SELECT trials (SELECT-NEXT, SELECT-BEYOND, SELECT-COMPARE, SELECT-MONOTHERAPY and SELECT-EARLY), with a maximum exposure of 2.5 years. It split the data into five data analysis sets: a short-term placebo group and long-term (i.e., up to 2.5 years) data for upadacitinib 15 mg QD, upadacitinib 30 mg QD, MTX censored at rescue and adalimumab also censored at rescue [63]. In this analysis, the frequency of adverse events is presented as exposure-adjusted event rates. Unless otherwise indicated, the data we present herein are based on this integrated safety analysis.

### 5.1. Overall Exposure and Tolerability/Safety Data

The integrated analysis included 3834 patients treated with upadacitinib (2630 with 15 mg QD and 1204 with 30 mg QD) for a mean duration of one year and totaling an exposure of 4020 patients/year (PY) (2655 PY with upadacitinib and 1365 PY with upadacitinib 30 mg QD). The total PY exposure for the comparators was 256.8 for placebo, 368.7 for MTX and 467.8 for adalimumab.

The most frequent (i.e., with ≥10 events per 100 PY (E/100 PY)) adverse events were upper respiratory tract infections, nasopharyngitis and urinary tract infections with both doses of upadacitinib and blood CPK increases with upadacitinib 30 mg QD.

With some limitations, the frequency of discontinuation due to adverse events and the frequency of serious adverse events are considered global or composite measures of tolerability and safety [64]. The incidence of adverse events leading to drug discontinuation with upadacitinib 15 mg QD (8.4 E/100 PY) was lower than that with upadacitinib 30 mg QD (13.3 E/100 PY) and no higher than that of adalimumab (11.1 E/100 PY), MTX (9.5 E/100 PY) or placebo (10.9 E/100 PY). A recent network meta-analysis of the efficacy and safety of JAKi (excluding filgotinib) and bDMARDs reported that, with the exception of certolizumab pegol and rituximab (which were associated with a higher frequency of discontinuations due to adverse events compared with placebo), none of the active drugs were significantly different from placebo regarding this outcome [55]. There were no differences between upadacitinib and baricitinib or tofacitinib in the likelihood of discontinuation due to adverse events [55].

The incidence of any serious adverse event was similar for upadacitinib 15 mg QD (15.0 E/100 PY) and adalimumab (15.6 E/100 PY) and higher than that of MTX (11.9 E/100 PY); upadacitinib 30 mg QD showed the highest incidence of serious adverse events (21.3 E/100 PY). The incidence of deaths was similar across the analyzed groups and the mortality rate for the upadacitinib groups was not different from that of the general population (standardized mortality ratio 0.58, 95% CI 0.37 to 0.85).

### 5.2. Infection

**Figure 4:**
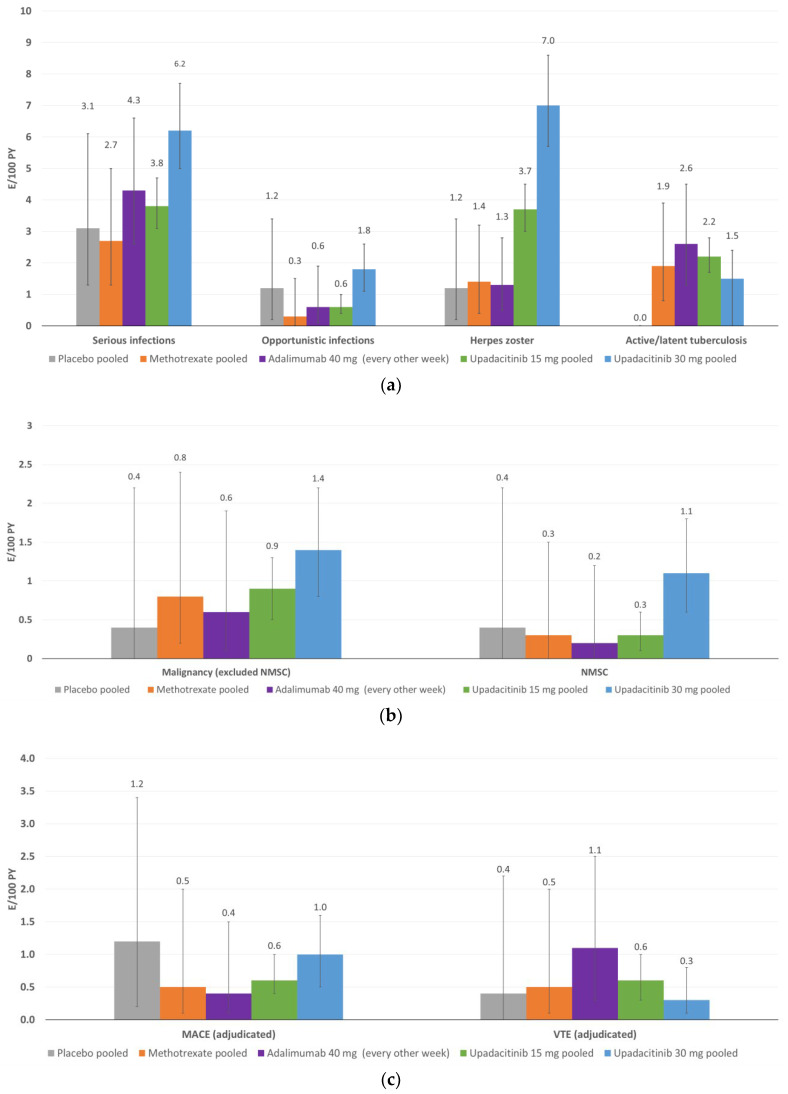

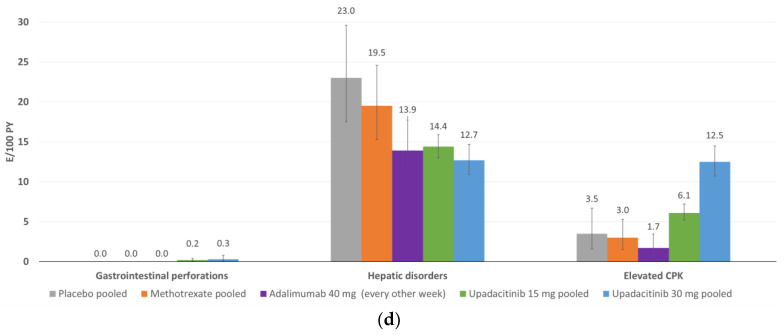
(**a**). Event rates for infections. (**b**). Event rates for malignancies. (**c**). Event rates for major adverse cardiovascular events and venous thromboembolism. (**d**). Event rates for other events of special interest: gastrointestinal perforation, hepatic disorders and CPK elevations. E/100 PY—events per 100 patient-years. NMSC—non-melanoma skin cancer. MACE—major adverse cardiovascular event; VTE—venous thromboembolic event. CPK—creatin phosphokinase.

The incidence of serious infections, opportunistic infections and active/latent tuberculosis was similar between upadacitinib 15 mg QD and adalimumab. However, the incidence of serious infections and opportunistic infections was higher in the 30 mg QD upadacitinib group (Figure 4a).

The incidence of herpes zoster was higher with upadacitinib than with placebo, adalimumab or MTX (Figure 4a). Over 90% of herpes zoster cases treated with upadacitinib were nonserious and more than 70% involved a single dermatome. A more recent multivariate analysis devoted to the identification of the risk factors for the occurrence of herpes zoster in upadacitinib-treated patients, using data from the six SELECT trials included in this review, found that a history of herpes zoster infection and Asian region vs. European region were associated with an increased risk of herpes zoster with both doses of upadacitinib, while female sex, older age and North American region were also predictive factors among patients receiving upadacitinib 15 mg [65]. In this latter analysis, coadministration of glucocorticoids or MTX was not associated with the occurrence of herpes zoster among patients treated with upadacitinib. An indirect comparison of tofacitinib, baricitinib and upadacitinib found that baricitinib was associated with the highest risk and upadacitinib with the lowest, although the uncertainty of the estimates does not allow us to make inferences regarding the comparative risk of the occurrence of herpes zoster with these three JAKi [66].

### 5.3. Malignancies

Patients with RA are at an increased risk of occurrence of certain types of cancer, such as lung cancer and lymphoma [67]. Therefore, it is important to understand the impact of new treatments for RA on the risk of malignancies.

In the integrated safety analysis of upadacitinib, the risk of nonmelanoma skin cancer (NMSC) and malignancies excluding NMSC was generally comparable across the analyzed groups, although the incidence was highest among patients treated with upadacitinib 30 mg QD (Figure 4b). The incidence of non-NMSC malignancies with upadacitinib 15 mg QD was not higher than expected in the US general population (an age- and gender-adjusted standardized incidence ratio of 1.05, 95% CI 0.66 to 1.60).

The use of bDMARDs combined with MTX in patients with rheumatoid arthritis appears to increase the risk of lymphoma and some solid tumors, such as breast, ovarian and lung cancers [68,69]. Regarding JAKi, a recent meta-analysis found no difference in the risk of NMSC and other malignancies, excluding NMSC, between patients treated with JAKi in combination with MTX and those treated with MTX alone [70].

### 5.4. Cardiovascular Events

The interest in the cardiovascular effects of JAKis lies in the fact that these drugs may reduce cardiovascular events and related deaths in patients with RA due to their anti-inflammatory properties, but they also have the potential for prothrombotic effects [71]. However, it is important to highlight that a recent noninferiority randomized trial (the ORAL Surveillance study) in a cardiovascular-risk enriched population of patients with RA failed to demonstrate the noninferiority of tofacitinib compared with a TNF inhibitor in terms of occurrence of adjudicated major adverse cardiovascular events (MACEs) and cancer, the coprimary endpoints, with a hazard ratio for MACEs of 1.33 (95%CI 0.91 to 1.94); the incidence of MACE in the combined doses (5 and 10 mg) of tofacitinib was 3.4% compared with 2.5% in the TNF inhibitor group [72].

The integrated safety analysis of the SELECT trials showed that the occurrence of major MACEs, which included cardiovascular death, nonfatal myocardial infarction and nonfatal stroke, was comparable across treatment groups (Figure 4c) and did not increase over time; similarly, the incidence of venous thromboembolic events was also comparable across treatment groups (Figure 4c). A systematic review and meta-analysis of the effect of DMARDs on MACEs that included five trials with JAKi (two with upadacitinib, two with baricitinib and one with filgotinib) showed that none of the DMARD subclasses (i.e., JAKi, TNF inhibitors and interleukin inhibitors) significantly affected the likelihood of occurrence of MACEs or that of myocardial infarction alone, stroke alone or all-cause mortality [73]. The results of that meta-analysis were consistent with those of a previous meta-analysis focused on JAKi that found no changes in the cardiovascular risk with these drugs among patients with RA [74]. A meta-analysis of JAKi in patients with immune-mediated inflammatory diseases found that there was no difference between JAKi and placebo in the risk of venous thromboembolism, pulmonary embolism or deep venous thrombosis [75]. Another meta-analysis had similar findings and suggested that the effect of JAKi on venous thromboembolism is not modified by the dose of drug, indication for treatment or length of follow-up [76]. However, it is also important to bear in mind the results of the ORAL Surveillance study that failed to demonstrate the noninferiority of tofacitinib compared with a TNF inhibitor in the occurrence of MACE [72].

### 5.5. Other Events of Special Interest

In the integrated safety analysis, gastrointestinal perforations were infrequently reported (two cases with upadacitinib 15 mg QD or <0.1 E/100 PY), with no cases in patients treated with placebo, MTX or adalimumab (Figure 4d). Although there are no comparisons with other JAKi, the incidence of gastrointestinal perforation with upadacitinib appears similar to that reported with other JAKi [77]. The incidence of hepatic disorders was similar between upadacitinib and adalimumab and lower versus MTX (Figure 4d).

With the exception of grade 3/4 decreases in hemoglobin and grade 4 lymphopenia that were more common with upadacitinib 30 mg QD, there were no substantial differences across treatment groups in the frequency of grade 3/4 decreases in hemoglobin, neutrophils and lymphocytes according to the integrated safety analysis of upadacitinib.

Finally, elevated CPK was more frequent with upadacitinib 15 mg QD and especially upadacitinib 30 mg QD compared with the other treatment groups (Figure 4d). Most of the cases were asymptomatic and few led to drug discontinuation.

### 5.6. To Date, Integrated Safety Analysis of Upadacitinib

An up-to-date safety analysis of the six SELECT trials included data from patients treated up to 4.5 years for a total exposure of 10,115 patients/years was recently reported as a poster at the EULAR European Congress of Rheumatology 2021 [78]. The results of this analysis were consistent with those mentioned above and the authors concluded that, with the exception of an increased frequency of herpes zoster infection and CPK elevation, the safety profile of upadacitinib 15 mg QD, including the incidence of serious infections, malignancies (excluding NMSC), MACEs and venous thromboembolism, was similar to that observed with adalimumab plus MTX.

A 3-year analysis of the safety data from the SELECT-COMPARE study, also presented as a poster at the EULAR European Congress of Rheumatology 2021 [79], showed a safety profile consistent with the results previously reported for the trial and with the integrated safety analysis. In this analysis, overall rates of death, malignancies, MACEs and venous thromboembolic events were reported were overall similar on upadacitinib and adalimumab with background MTX therapy.

## 6. A Clinician’s Perspective on Upadacitinib

The efficacy of upadacitinib, a preferential JAK 1 inhibitor, has been demonstrated in an extensive clinical program in different RA populations (SELECT trials), MTX-naïve, csDMARD failure and biological refractory patients. In all of these RA populations, upadacitinib achieved the primary endpoints for efficacy and most of the secondary confirmatory outcomes, supporting the efficacy of this JAK 1 targeted drug in RA. Efficacy was demonstrated either in combination therapy (with MTX or with other csDMARDs) or in monotherapy regimens. The safety profile was also analyzed in the randomized phases and in the open extension periods of these clinical trials, with no evidence of new relevant safety alerts. In view of the results of the SELECT program, upadacitinib has recently received approval for the treatment of RA by the EMA and FDA. It, together with tofacitinib and baricitinib, is currently included in the available targeted synthetic therapies for RA. More recently, the EMA approved filgotinib, another preferential JAK1 inhibitor for RA treatment.

The results of the efficacy of upadacitinib in the SELECT program are highly consistent, showing high remission rates in the different RA populations regardless of the remission criteria used [80]. However, a relevant question arises in view of the recently released results from the phase III randomized clinical trials: is the efficacy of upadacitinib superior or at least similar to that observed with other approved jakinibs such as baricitinib, tofacitinib or filgotinib? Although the chemical structure, JAK selectivity and pharmacodynamics differ among jakinibs, there is currently no robust answer to that clinical question due to the lack of head-to-head randomized clinical trials comparing jakinibs. In a network meta-analysis of 11 randomized clinical trials in RA patients who had failed to develop csDMARDS, the efficacy of tofacitinib, baricitinib and upadacitinib was compared [52]. There were no significant differences in the response rates among jakinibs, although numerically higher ACR20 and ACR50 response and remission rates were observed with upadacitinib 15 mg QD in comparison with the other jakinibs. Similar efficacy results were observed in a network meta-analysis of randomized clinical trials of the four jakinibs and with peficitinib when used as monotherapy [51]. In a recent network meta-analysis of randomized clinical trials in patients who were nonresponders to tsDMARDs such as tofacitinib, baricitinib, upadacitinib and filgotinib [53], no definitive conclusions can be drawn, since the response rates may have some limited differences depending on the ACR response criteria used. Therefore, the indirect comparative efficacy between upadacitinib and other jakinibs should be interpreted cautiously.

It is important to highlight that the efficacy of upadacitinib plus MTX was compared in head-to-head randomized clinical trials with biological therapies, demonstrating superiority over adalimumab plus MTX in patients after csDMARD failure [23]. Similar head-to-head randomized comparisons were performed in this RA population with tofacitinib [81], baricitinib [30] and filgotinib [82]. In these trials, only baricitinib showed superiority over adalimumab. To our knowledge, there is only one randomized head-to-head trial comparing a biological drug to a jakinib in patients who failed to respond to a first biological therapy: the SELECT-CHOICE trial [24]. In this trial, upadacitinib showed superiority compared with abatacept in terms of mean change from baseline in the DAS28-CRP values and clinical remission according to DAS28-CRP of <2.6. Although these results should not be extrapolated to other JAKi or other biologics (TNF antagonists or non-TNF inhibitors), this fact reinforces the robust efficacy of this drug in both RA populations.

An interesting and relevant impact on some PRO, such as pain, has been observed in patients treated with upadacitinib; whether this improvement in the efficacy may be due in part to an effect of a noninflammatory component is not well understood. In the SELECT-COMPARE clinical trial [23], the mean differences in the number of swollen joint counts across the different visits in the two arms (upadacitinib and adalimumab) were not as prominent as the other efficacy measures, although, in a post hoc analysis, the proportion of patients without swollen joints was higher in patients treated with upadacitinib [83]. Similar findings were observed in SELECT-CHOICE (upadacitinib vs. abatacept), where no differences were observed in the number of swollen joint counts between arms, but differences were observed in the other components of the DAS28 score (i.e., CRP, tender joint count and patient global assessment) [24]. This particular additional beneficial effect not related to an improvement in inflammation (synovitis) is interesting and warrants further investigation. Whether this is a characteristic of upadacitinib compared with other jakinibs is not known at this time, although similar effects were also observed with other jakinibs, suggesting a possible family class effect [84].

One of the notable points in the clinical response to upadacitinib in the different clinical trials is the similar rates of clinical response, including the remission rates, regardless of the population analyzed (naïve, MTX-failure or biologic failure). Even after a biological failure, the response is similar, regardless of the number of previous biologics [85]. This finding is of interest and is different from the biologic drugs and firmly addresses the efficacy of this drug in refractory populations. This particular clinical benefit also seems to be observed with other JAKi [86].

Another relevant clinical issue to elucidate is the efficacy of switching between JAKi. Real-world data on this topic are scarce, but one preliminary US study showed similar clinical improvement with upadacitinib in patients with or without prior tofacitinib [61]. These initial results are interesting but should be confirmed in prospective registries with a significant number of patients to draw a solid conclusion.

The safety profile of upadacitinib is generally consistent with those observed with biological or other JAK inhibitors, with the exception of a higher frequency of some events seen with JAKi, such as herpes zoster and CPK elevations. The rate of some adverse events was lower in patients treated with the low dose (15 mg QD) vs. the high dose (30 mg QD); however, the 30 mg QD dose is not approved for the treatment of RA [63]. Taking into account the warnings for a pan-JAK inhibitor (tofacitinib) focusing on major cardiovascular events and malignancies as a consequence of a single comparative safety trial with TNF inhibitors and the recent results of the ORAL Surveillance study commented above, it is quite important to confirm these adverse events and to define whether they are drug-specific or class-specific (JAK inhibition) [72]. Integrated safety data of the clinical trials in RA accounting for 4020 patients/year of exposure did not find a significant increase in these adverse events with upadacitinib [63]. However, only real-world data with prospective observational studies with a long-term follow-up could provide a more robust answer to this or other questions, such as whether some adverse events may be avoided with a selective JAK1 inhibitor in comparison with a more pan-JAK inhibition.

In conclusion, upadacitinib (a preferential JAK1 inhibitor) has emerged as a new therapeutic drug with sustained efficacy and has demonstrated a good safety profile for treating patients with RA.

## Figures and Tables

**Table 1:** Main pharmacokinetic characteristics of extended upadacitinib release.

Characteristic *		
**Absorption**	t_max_, median: 2–4 h	
**Distribution**	Protein binding 52%	
**Metabolism**	Mainly CYP3A4 and minor contribution of CYP2D6	
**Elimination**	Predominantly as the unchanged parent substance in urine (24%) and feces (38%).	
	Terminal elimination half-life; mean: 9–14 h	
**Intrinsic factor**	**Effect**	**Recommendation**
Age, sex, body weight, race and ethnicity	No clinically meaningful effect on upadacitinib exposure	No dose adjustment of upadacitinib is warranted based on these characteristics
Renal impairment	Upadacitinib AUC was 18%, 33% and 44% higher in subjects with mild, moderate and severe renal impairment, respectively, compared with subjects with normal renal function. Upadacitinib C_max_ was similar in subjects with normal and impaired renal function.	No dose adjustment is required in patients with mild or moderate renal impairment. The recommended dose is 15 mg once daily for patients with severe renal impairment.
Hepatic impairment	Upadacitinib AUC was 28% and 24% higher in subjects with mild and moderate hepatic impairment, respectively, compared with subjects with normal liver function. Upadacitinib C_max_ was unchanged in subjects with mild hepatic impairment and 43% higher in subjects with moderate hepatic impairment compared with subjects with normal liver function.	No dose adjustment is required in patients with mild (Child–Pugh A) or moderate (Child–Pugh B) hepatic impairment.
	Upadacitinib was not studied in patients with severe (Child–Pugh C) hepatic impairment	Upadacitinib should not be used in patients with severe (Child–Pugh C) hepatic impairment.

* Based on the information provided in the summary of product characteristics of upadacitinib [5].

**Table 2:** Main characteristics of the trials included in the phase 3 SELECT program with upadacitinib.

	Inadequate Response to DMARDs					Naïve Patients
	NEXT	BEYOND	COMPARE ^§^	CHOICE	MONOTHERAPY	EARLY
Design	12-wk, M, R, DB	12-wk, M, R, DB	26-wk, M, R, DB	24-wk, M, R, DB	14-wk, M, R, DB	48-wk, M, R, DB
Population	csDMARD-IR	bDMARD-IR	Methotrexate -IR	bDMARD-IR	Methotrexate -IR	Naïve or limited exposure to methotrexate
Background therapy	csDMARD	csDMARD	Methotrexate	csDMARD	Not applicable	Not applicable
Upadacitinib arms	15 mg QD 30 mg QD	15 mg QD 30 mg QD	15 mg QD	15 mg QD	15 mg QD 30 mg QD	15 mg QD 30 mg QD
Comparator	Placebo	Placebo	Placebo Adalimumab 40 mg/2 wk	Abatacept	Methotrexate	Methotrexate
Type of treatment	Combination	Combination	Combination	Combination	Monotherapy	Monotherapy
Sample size	661	499	1629	612	648	947
Primary endpoint	ACR20 at wk 12 DAS28-CRP ≤ 3.2 at wk 12	ACR20 at wk 12 DAS28-CRP ≤ 3.2 at wk 12	ACR20 at wk 12 DAS28-CRP < 2.6 at wk 12	∆DAS28-CRP at week 12 (noninferiority)	ACR20 at wk 14 DAS28-CRP ≤ 3.2 at wk 14	ACR50 at wk 12 DAS28-CRP < 2.6 at wk 24
Confirmatory endpoints (FDA)	∆DAS28-CRP ∆HAQ-DI ∆SF-36 PCS DAS28-CRP ≤ 3.2 DAS28-CRP < 2.6 CDAI ≤ 10 ∆Morning stiffness duration ∆FACIT-F	∆DAS28-CRP ∆HAQ-DI DAS28-CRP ≤ 3.2 ∆SF-36 PCS	∆DAS28-CRP ∆mTSS ∆HAQ-DI ACR50 | ∆SF-36 PCS DAS28-CRP ≤ 3.2 DAS28-CRP < 2.6 CDAI ≤ 10 ∆FACIT-F ∆Morning stiffness duration ACR50 ¶ ∆HAQ-DI ¶ ∆ Pain ¶	∆DAS28-CRP at week 12 (superiority) DAS28-CRP < 2.6 at week 12 (superiority)	∆DAS28-CRP ∆HAQ-DI ∆SF-36 PCS DAS28-CRP ≤ 3.2 DAS28-CRP < 2.6 ∆Morning stiffness duration	∆HAQ-DI ∆mTSS † DAS28-CRP ≤ 3.2 DAS28-CRP < 2.6 † ∆SF-36 PCS
Confirmatory endpoints (EMA)	∆DAS28-CRP ∆HAQ-DI ACR20 ∆SF-36 PCS DAS28-CRP < 2.6 CDAI ≤ 10 ∆Morning stiffness duration ∆FACIT	∆DAS28-CRP ACR20 ∆HAQ-DI ∆SF-36 PCS	∆mTSS DAS28-CRP ≤ 3.2 ∆DAS28-CRP ∆HAQ-DI ACR20 DAS28-CRP ≤ 3.2 | ∆SF-36 PCS CDAI ≤ 10 ∆Morning stiffness duration ∆FACIT mTSS ≤ 0 ‡		∆DAS28-CRP ∆HAQ-DI ∆SF-36 PCS DAS28-CRP ≤ 3.2 DAS28-CRP < 2.6 ∆Morning stiffness duration	∆DAS28-CRP † ∆HAQ-DI † ACR50 † ∆mTSS † DAS28-CRP ≤ 3.2 † ∆SF-36 PCS † % no radiographic progression: mTSS ≤ 0 †

ACR20—American College of Rheumatology 20% improvement; ACR50—American College of Rheumatology 50% improvement; bDAMRD-IR—inadequate response to biologic disease-modifying antirheumatic drugs; CDAI—clinical disease activity index; csDMARD—conventional synthetic disease-modifying antirheumatic drugs; csDMARD-IR—inadequate response to conventional synthetic disease-modifying antirheumatic drugs; ∆—mean change from baseline; DAS28-CRP—disease activity score in 28 joints using C-reactive protein level; DB—double-blind; DMARDs—disease-modifying antirheumatic drugs; FACIT-F—functional assessment of chronic illness therapy fatigue scale; HAQ-DI—health assessment questionnaire disability index; M—multinational; mTSS—modified total Sharp/van der Heijde score; TX-IR—inadequate response to methotrexate; OD—once daily; R—randomized; SF-36 PCS—36-item short form health survey physical component summary score; wk—week. All endpoints were assessed at week 12 unless stated otherwise. All primary and ranked secondary endpoints were multiplicity controlled and met statistical significance denoted by a *p* value of ≤0.05. † assessment at week 24; ‡ assessment at week 26; § all comparisons were for upadacitinib + methotrexate vs. placebo + methotrexate, unless indicated as follows, | adalimumab + methotrexate (non-inferiority); ¶ adalimumab + methotrexate (superiority).

**Table 3:** Primary efficacy results of the superiority clinical trials from the SELECT program with upadacitinib.

Study Name	Population	Primary Endpoints	UPA 15 mg	UPA 30 mg	PBO	MTX	ADA	ABA	Conclusion
NEXT [21]	csDMARD-IR	ACR20 at wk 12	64% ^‡^	66% ^‡^	36%	-	-		Both doses of UPA QD were superior to placebo.
		DAS28-CRP ≤ 3.2 at wk 12	48% ^‡^	48% ^‡^	17%	-	-		
BEYOND [22]	bDMARD-IR	ACR20 at wk 12	65% ^‡^	56% ^‡^	28%	-	-		Both doses of UPA QD were superior to placebo.
		DAS28-CRP ≤ 3.2 at wk 12	43% ^‡^	42% ^‡^	14%	-	-		
COMPARE [23]	Methotrexate -IR	ACR20 at wk 12	71% ^†,ǀ^	-	36%	-	63%		UPA 15 mg QD was superior to adalimumab
		DAS28-CRP < 2.6 at wk 12	29% ^†,¶^	-	6%	-	18%		
CHOICE [24]	bDMARD-IR	Change from baseline in the DAS28-CRP (non-inf.)	−2.52 points ^‖^					−2.00 points	UPA 15 mg QD was superior vs. abatacept
MONOTHERAPY [25]	Methotrexate -IR	ACR20 at wk 14	68% ^¶¶^	71% ^¶¶^	-	41%	-		Both doses of UPA QD were superior to continuing MTX
		DAS28-CRP ≤ 3.2 at wk 14	45% ^¶¶^	53% ^¶¶^	-	19%	-		
EARLY [26]	Naïve or limited exposure to methotrexate	ACR50 at wk 12	52% ^¶^	56% ^¶^		28%			Both doses of UPA QD were superior to MTX
		DAS28-CRP < 2.6 at wk 24	48% ^¶^	50% ^¶^		19%			

ACR20—American College of Rheumatology 20% improvement; ACR50—American College of Rheumatology 50% improvement; ABA—abatacept; ADA—adalimumab; bDAMRD-IR—inadequate response to biologic disease-modifying antirheumatic drugs; csDMARD—conventional synthetic disease-modifying antirheumatic drugs; csDMARD-IR—inadequate response to conventional synthetic disease-modifying antirheumatic drugs; DAS28-CRP—disease activity score in 28 joints using C-reactive protein level. DAS28 < 2.6 reflects clinical remission and DAS28 < 3.2 reflects low disease activity; MTX—methotrexate; PBO—placebo, QD—once daily; UPA—upadacitinib. ^‡^ *p* < 0.0001 for upadacitinib versus placebo. ^†^ *p* < 0.001 for upadacitinib + methotrexate versus placebo + methotrexate. ^ǀ^ *p* < 0.05 for upadacitinib + methotrexate versus adalimumab + methotrexate. ^‖^ *p* < 0.001 for noninferiority versus abatacept. ^¶^ *p* < 0.001 for upadacitinib + methotrexate versus adalimumab + methotrexate (COMPARE), upadacitinib vs. methotrexate (EARLY). ^¶¶^ *p* < 0.0001 for upadacitinib versus methotrexate.

**Table 4:** Results of upadacitinib on key patient-reported outcomes in the SELECT trial program.

Study	Outcome ^a^	Week	Placebo	MTX	ABA	ADA	Upa 15 mg	Upa 30 mg
NEXT [21]	ΔVAS Pain	12	−10.3				−29.9 ^‡^	−31.7 ^‡^
	ΔFACIT-F	12	3.0				7.9 ^‡^	7.7 ^‡^
	ΔSF36-PCS	12	3.0				7.6 ^‡^	8.0 ^‡^
	ΔHAQ-DI	12	−0.26				−0.61 ^‡^	−0.55 ^‡^
BEYOND [22]	ΔSF36-PCS	12	2.4				5.8 ^‡^	7.0 ^‡^
		24	NA				7.2	8.0
	ΔHAQ-DI	12	−0.16				−0.41 ^‡^	−0.44 ^‡^
COMPARE [23]	ΔVAS Pain	12	−15.7			−25.6	−32.1 ^†,¶^	
	ΔFACIT-F	12	4.8			7.4	9.0 ^†,ǀ^	
		26	5.5			8.2	9.7 ^†,ǀ^	
	ΔSF36-PCS	12	3.6			6.3	7.9 ^†,ǁ^	
		26	4.5			7.8	9.5 ^†,ǁ^	
	ΔHAQ-DI	12	−0.28			−0.49	−0.60 ^†,ǁ^	
CHOICE [24]	ΔFACIT-F	12			8.35		9.61	
		24			10.32		10.73	
	ΔSF36-PCS	12			7.03		9.62	
		24			9.40		10.97	
MONOTHERAPY [25]	ΔVAS Pain	14		−13.88			−26.15 ^¶¶^	−33.18 ^¶¶^
	ΔSF36-PCS	14		4.3			8.3 ^¶^	10.2 ^¶^
	ΔHAQ-DI	14		−0.32			−0.65 ^¶^	−0.73 ^¶^
EARLY [26]	ΔVAS Pain	12		−25.36			−36.28 ^¶^	−39.67 ^¶^
		24		−28.40			−39.84 ^¶^	−44.67 ^¶^
	ΔFACIT-F	12		6.80			10.01 ^¶^	9.57 ^¶^
		24		7.37			10.59 ^¶^	10.56 ^¶^
	ΔSF36-PCS	12		5.77			10.09 ^¶^	10.11 ^¶^
		24		6.99			10.97 ^¶^	11.69 ^¶^
	ΔHAQ-DI	12		−0.49			−0.83 ^¶^	−0.86 ^¶^
		24		−0.60			−0.87 ^¶^	−0.91 ^¶^

^a^ Minimum clinically important differences: VAS pain, ≥10 mm; FACIT-T, ≥4 points; SF36-PCS, ≥2.5 points; HAD-DI, ≥0.22 units. ADA—adalimumab; ABA—abatacept; ∆—mean change from baseline; FACIT-F—functional assessment of chronic illness therapy fatigue scale; HAQ-DI—health assessment questionnaire disability index; MTX—methotrexate; SF-36 PCS—36-item short form health survey physical component summary score; VAS—visual analog scale. ^†^ 0.001 upadacitinib versus placebo. ^‡^ 0.0001 upadacitinib versus placebo. ^ǀ^ 0.05 upadacitinib versus active comparator (adalimumab, abatacept, methotrexate). ^ǁ^ 0.01 upadacitinib versus active comparator. ^¶^ 0.001 upadacitinib versus active comparator. ^¶¶^ 0.0001 upadacitinib versus active comparator.

## Data Availability

Data sharing is not applicable to this article as no datasets were generated or analyzed for this manuscript.
